# Pycnogenol a promising remedy for diabetic keratopathy in experimentally induced corneal alkali burns in diabetic rats

**DOI:** 10.1186/s12917-022-03307-3

**Published:** 2022-05-30

**Authors:** Mohamed A. Hamed, Amany Farag, Ibrahim S. Zahran, Ahmed Hafez, Mohamed Abdo Rizk, Marwa Abass

**Affiliations:** 1 Department of Surgery, Anesthesiology, and Radiology, Faculty of Veterinary Medicine, Aswan University, Aswan, Egypt; 2 Department of Cytology and Histology, Faculty of Veterinary Medicine, Mansoura University, Mansoura, 35516 Egypt; 3 Department of Physiology, Faculty of Veterinary Medicine, Aswan University, Aswan, Egypt; 4 Department of Pharmacology, Faculty of Veterinary Medicine, Aswan University, Aswan, Egypt; 5 Department of Internal Medicine, Infectious and Fish Diseases, Faculty of Veterinary Medicine, Mansoura University, Mansoura, 35516 Egypt; 6 Department of Surgery, Anesthesiology, and Radiology, Faculty of Veterinary Medicine, Mansoura University, Mansoura, 35516 Egypt

**Keywords:** Pycnogenol, Cornea, Epithelium, Diabetes, Wound healing

## Abstract

**Aim:**

This study aimed to investigate the efficiency of topically applied pycnogenol (PYC) in healing the standardized alkaline corneal ulcer in diabetic and normal rats.

**Materials and methods:**

The corneal alkali-burn injury (CA-I) model was unilaterally developed in Wistar rats by filter paper saturated with 0.01 M of NaOH and touching the eyes for 45 s. Rats were divided into four groups: Normal control (NC), normal PYC (NPYC), diabetic control (DC), and diabetic PYC (DPYC). Both NPYC and DPYC groups were daily treated with PY eye drops three times, whereas NC and DC ones were treated with ordinary saline for six successive days.

**Results:**

The wound healing of corneal epithelial was improved in the NPYC group compared to the NC group. Meanwhile, it was significantly improved (*P* < 0.05) in the DPYC group than in the DC group. Histological examination revealed that corneal re-epithelialization was more accomplished in the DPYC group than in the DC group. In addition, the inflammatory cells were augmented in the DC group more than those in the DPYC one.

**Conclusion:**

The findings obtained revealed the efficiency of PYC for enhancing the corneal re-epithelialization and reducing the inflammatory reaction post alkali burn in rats, and thus it could be beneficially valuable as a treatment for the diabetic keratopathy.

## Background

Corneal alkali injuries (CA-I) are common in veterinary ophthalmology and have a significant clinical problem, resulting in vision impairment. The causes of superficial corneal ulceration include trauma, spontaneous chronic corneal epithelial defects, aberrant hairs, i.e., distiches, ectopic cilia, and keratoconjunctivitis sicca [1]. CA-I is characterized by the massive infiltration of polymorphonuclear leucocytes into the stroma and severe destruction of corneal collagen [2]. Additionally, CA-I induces denaturation of the cornea’s anterior layers and stroma, which may induce fibroblastic activity and disorganized collagen, thereby forming corneal scarring and neovascularization [3].

Corneal complications (diabetic keratopathy) are generally correlated with diabetes mellitus (DM), and these complications are related to hyperglycemia levels [4, 5]. Diabetic keratopathy has been estimated to occur in more than 50% of diabetic patients during their disorder. Diabetic keratopathy-related corneal disorders include nonhealing epithelial defects, leading to infections, corneal ulcers, secondary scarring, and permanent vision loss [5]. Conventional therapy approaches are ineffective against these epithelial abnormalities (e.g., lubricants, antibiotics, bandage contact lens, and tarsorrhaphy) [6]. These treatments do not accelerate the corneal epithelial wound curative but rather protect the corneal surface from the exterior stimuli until the wound heals completely. However, organ cultured-corneas are the preferable treatment to accelerate corneal epithelial migration and mitosis in the eyes [7]. Therefore, the main purpose of management of corneal ulcer was to stimulate corneal epithelial migration and prevent bacterial multiplication of the exposed corneal stroma until epithelium healing occurs.

Numerous trials have been conducted to improve the corneal wound healing (CWH) via topically applied medications, including ascorbate [8], fibronectin [1], sodium hyaluronan [3], metalloproteinase inhibitor GM6001 [9], epidermal growth factor [10], amniotic membranes [11], bovine amniotic fluid [12], and *Aloe vera* [13]. In this regard, pycnogenol (PYC) played a crucial role in wound curative [14]. PYC is a standardized extract from French maritime pine bark (*Pinus pinaster*) containing proanthocyanins as a herbal remedy, nutrition, and supplemental food for many degenerative diseases. PYC has photoprotective, antioxidant, antimicrobial, and anti-inflammatory effects [15] with extreme affinity to elastin and collagen and could mitigate the enzymatic hydrolysis via matrix metalloproteinases, accelerating the wound healing [16, 17] since DM is related to the oxidative stress that reduces the wound healing process [18]. At the same time, no data on the effect of PYC on CWH in diabetic patients have been reported. It was hypothesized that utilization of PYC would accelerate the wound healing process in the diabetic condition. Consequently, the present experimental study aims to investigate the curative efficacy of PYC on CWH in normal and diabetic rats.

## Results

### PYC accelerates the healing rate of corneal ulcer

**Fig. 1:**
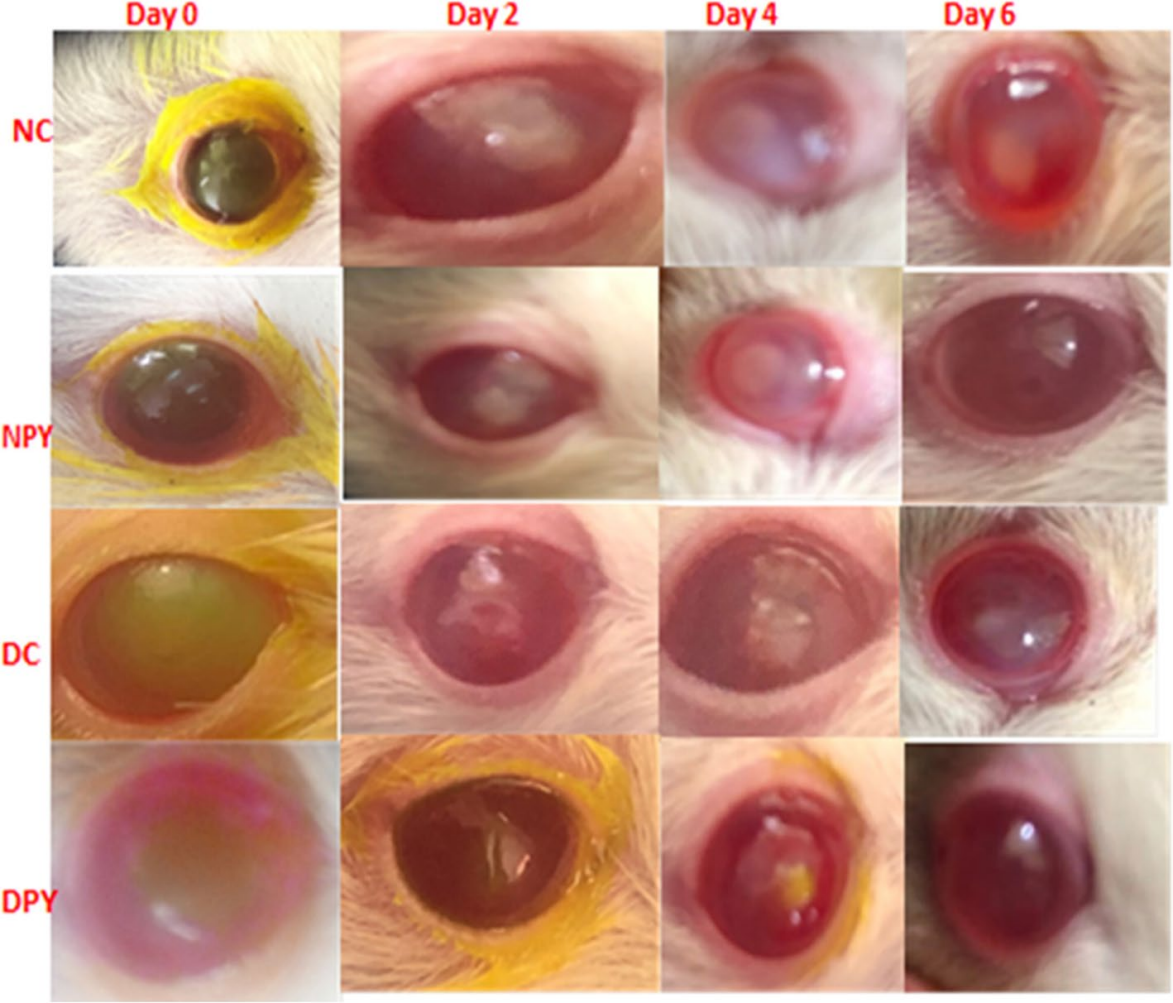
Corneal wound healing in normal and diabetic rats treated with pycnogenol. Photographs of fluorescein staining of corneal epithelial defects in NC, NPYC, DC, and DPYC groups at 0-, 2-, 4-, and 6-days post wounding. Photographs of fluorescein staining revealed that the NPYC group had better-wound healing than the NC group at 2-, 4- and 6-days post wounding, while DPYC showed smaller corneal epithelial defect than DC at 2-, 4- and 6-days post wounding. NC (normal control), NPYC (normal pycnogenol), DC (diabetic control) and DPYC (diabetic pycnogenol)

Before the experiment, all rats underwent a comprehensive ophthalmic investigation containing slit-lamp biomicroscopy, indirect ophthalmoscopy, fluorescein staining, and Schirmer’s tear test. The corneal injury areas stained with fluorescein were evaluated to express the wound defect area’s size at 0-, 2-, 4-, and 6-d post wounding (Fig. 1). There was no significant difference in wound size between non-diabetic and diabetic rats immediately after initiating burn wound injury (Fig. 1). Epithelial healing was significantly slowed (*P < 0.05*) throughout the first 2 days after wounding in control and diabetic control rats compared with the diabetic PYC (DPYC)-treated rats. The mean healing rate of the diabetic control (DC)-group was significantly lower than that of the normal control (NC)-groups at 2-, 4-, and 6-d post wounding (*P < 0.05*) (Table 1), while the healing rate was significantly higher in the normal PYC (NPYC)-groups than in the NC-group throughout the 6-day study period (*P* < 0.05) (Table 1). The healing rate of DPYC was significantly higher than in the DC group throughout the 6-day study period (*P < 0.05*) (Table 1). Throughout the study period, no significant differences (*P* > 0.05) were detected between NPYC and DPYC groups. Compared to other groups, the corneal epithelial defects of the NPYC and DPYC groups had completely re-epithelialized 6 days after wounding (Fig. 1).

**Table 1:** Healing rate of pycnogenol on corneal alkali burns in normal control, normal diadetic, diabetic control and diabetic pycnogenol rats

Groups	Day 0	Day 2	Day 4	Day 6
**NC**	0	11.5 ± 2.54^a^	36.33 ± 3.93^a^	81.83 ± 1.57^a^
**NPYC**	0	34.05 ± 2.48^b^	63.92 ± 1.96^b^	99.00 ± 0.98^b^
**DC**	0	7.67 ± 2.04^a^	25.67 ± 3.14^a^	63.87 ± 1.89^a^
**DPYC**	0	33.87 ± 2.51^b^	63.28 ± 1.22^b^	97.33 ± 2.25^b^

^a, b^: medians and ranges with different superscript letters at the same column are significantly different at *P < 0.05*. Normal control (NC), normal PYC (NPYC), diabetic control (DC), and diabetic PYC (DPYC)

### PYC enhanced the corneal re-epithelialization and reduced the inflammatory reaction post alkali burn

**Fig. 2:**
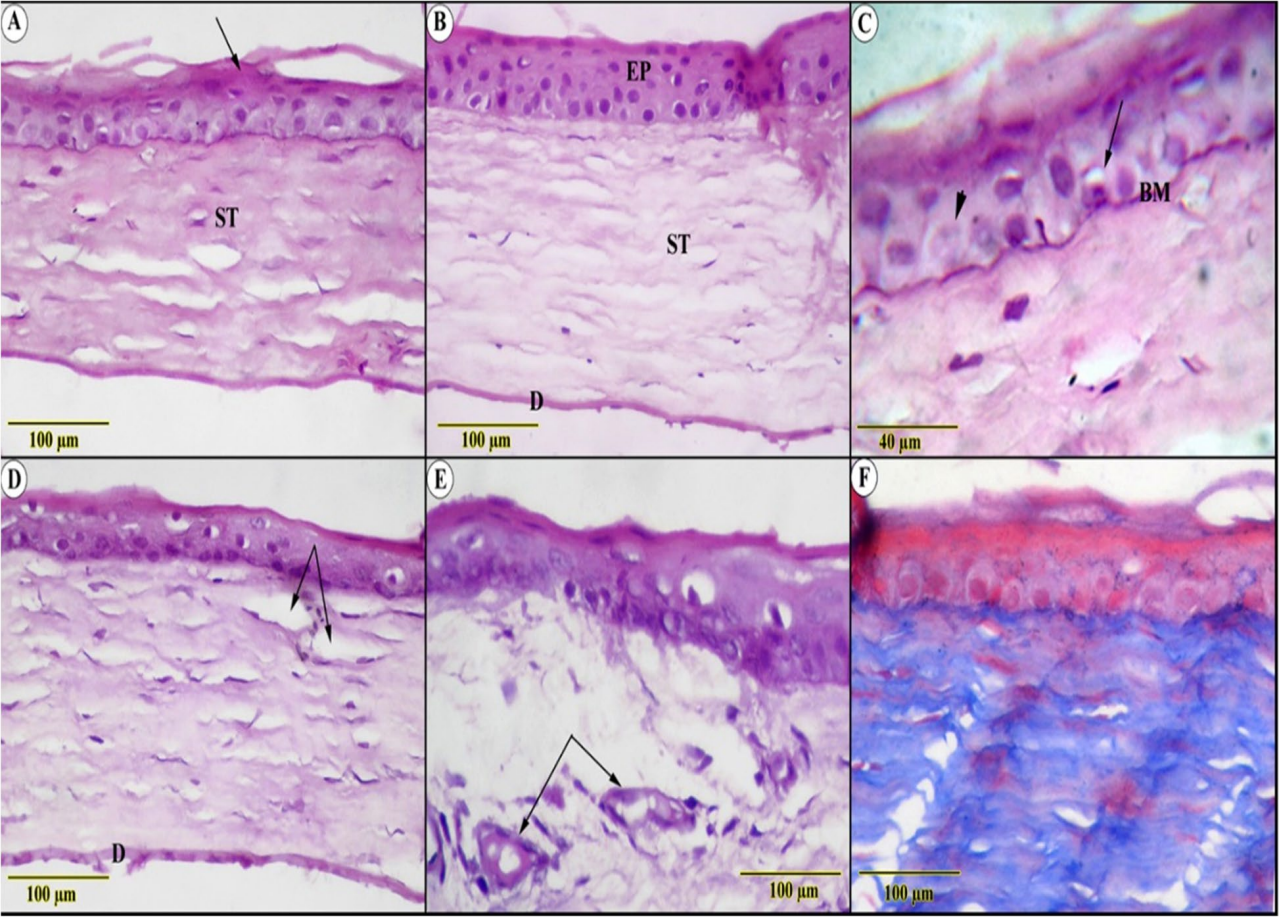
Histological examination in ulcerated (**A**, **C**, **D**, **E**, **F**) and control (B) sections showed (**A**) thinning of the corneal epithelial with keratosis (arrow) above the edematous stroma (ST), (**B)** normal architecture of the multilayered non-keratinized epithelium (EP), parallel arranged stroma (ST) and Descement’s membrane (D) (**C**) high magnified section showed karyolytic (arrowhead) and pyknotic (arrow) nuclei of the basal cells resting on a wavy basement membrane (BM) (**D**&**E**) edematous collagenous stroma (ED) surrounding small blood vessels and leukocytic infiltration (arrows) (**F**) blue-stained immature granulation tissue (Trichrome stain)

In the ulcerated group; one of the distinguished observations was the erosion of intermediate polyhedral layers that displayed the epithelium as 2–3 layers contrary to the 6-layers of normal tissues. As well as the notable keratosis that overwhelmed the superficial squamous layer (Fig. 2A, B). The impact of the alkali on the cellular level was manifested by vacuolated cytoplasm, in addition to nuclear changes in the form of hyperchromasia, pyknosis, and karyolysis. The effect extends to both the basement and Bowman’s membranes, where they appear wavy in shape and partially segmented (Fig. 2C). The modulation that occurred on the edematous stroma, where its lamellae were widely separated in between the scattered large, irregularly shaped keratocytes, could be linked to the opacity in the macroscopic figures (Fig. 2D). The examination also revealed evidence of leukocytic infiltration, though the most significant change was the neovascularization in avascular tissue (Fig. 2E). Trichrome staining was used to identify faintly blue-colored collagen fibers, which were thought to be evidence of auto-regeneration of stromal-immature granulation tissue (Fig. 2F). The Descement’s membrane appears thinned out and fragmented collateral with attenuation of the endothelial cells.

**Fig. 3:**
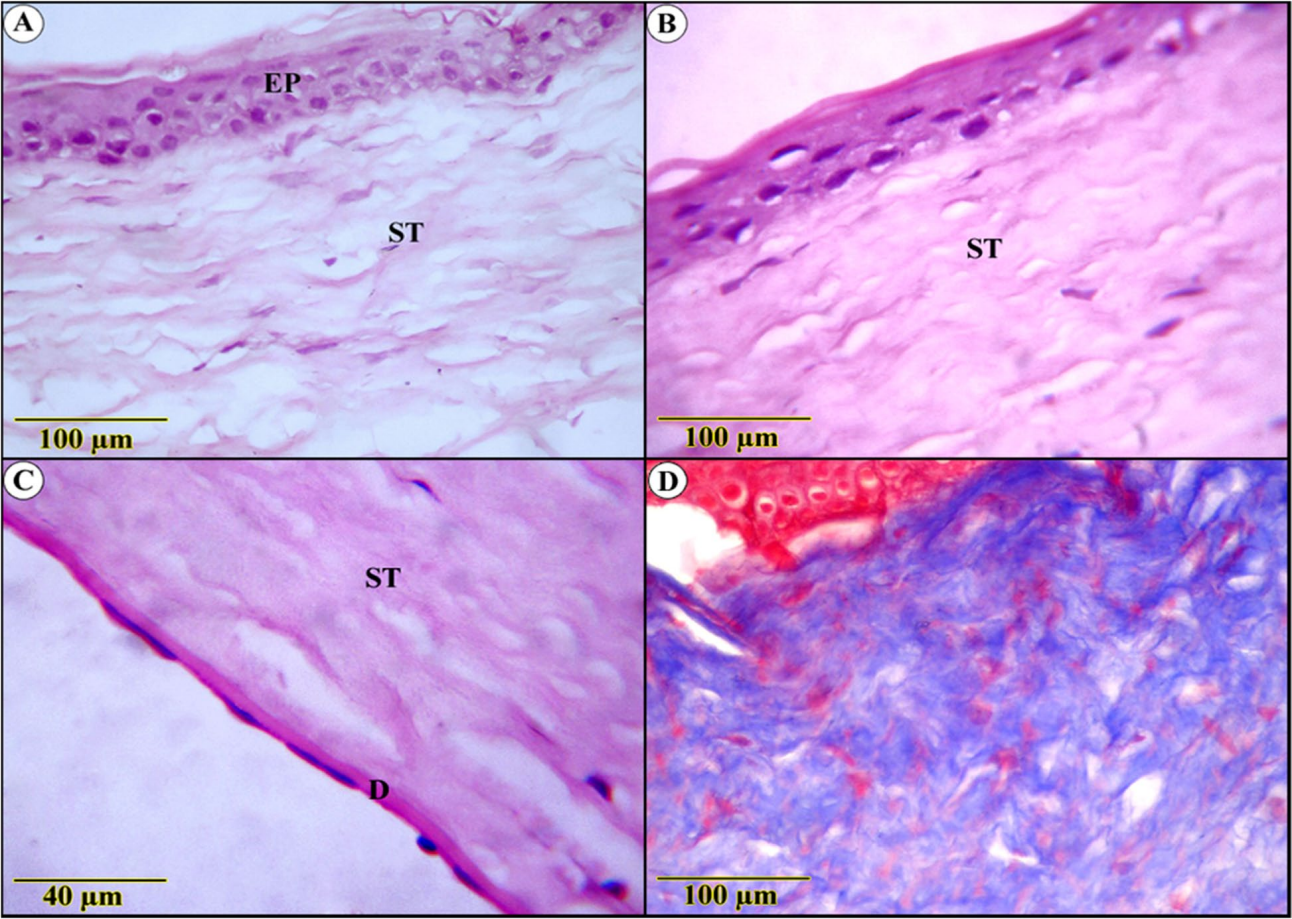
Histological examination in Pycnogenol-treated ulcer sections (**A**, **B**, **C**, **D**) show (**A**) apparent renewal of the multilayered epithelium covered with superficial flattened cells (EP) and mild keratosis (arrow), proliferative stroma with few spaces in between (ST), (**C**) Magnified section showed organized stroma supported by the Descement’s membrane and the endothelium (**D**) blue-stained parallel lamellae of regular collagenous fibers (Trichrome stain)

The re-construction of shattered layers in PYC-treated ulcer sections was so remarkable that it was difficult to distinguish them from uninjured tissues (Fig. 3A). However, slight keratosis and a few degenerative nuclear changes were detected (Fig. 3B). The stromal lamellae displayed highly proliferative activity concomitantly lacking disruption among the collagen fibers induced in injured specimens, along with notable regression of immature collagen fibers and accretion of mature ones (Fig. 3C). Descemet’s membrane was observed to be a thick homogenous non-cellular membrane, while the endothelium exhibited an ordinary structure (Fig. 3D).

**Fig. 4:**
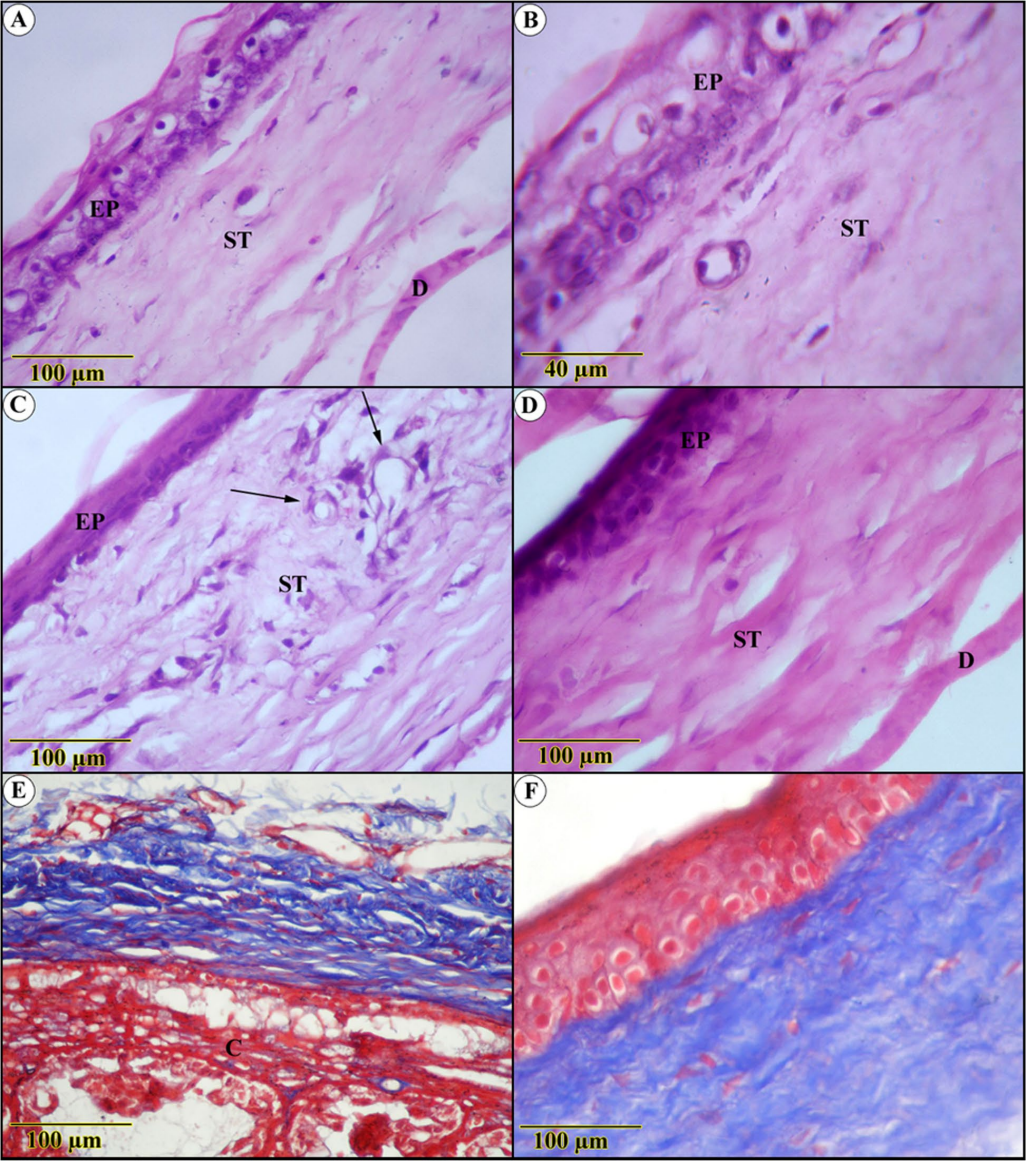
Histological examination in diabetes-inducing to ulcerated rats group (**A**, **B**, **C**, **D**, **E**) and control (**F**) group show (**A** and **B**) severe degenerative changes of both epithelium (EP) and the underlying stroma (ST) (**C**) blood vessels and leukocytic infiltration (**D**) fibrotic stroma with thickened Descement’s membrane (**E**) Densely packed blue-stained collagen bundles, ciliary body hyperplasia(C) (**F**) highly ordered blue-stained lamellae of collagenous fibers (Trichrome stain)

Compared to diabetic-free rats, the influences on diabetes-inducing ulcerated rats were aggressive. This hypothesis was based on a significant decrease in epithelial thickness, to the point where some segments revealed only basal layer reality. Nonetheless, it demonstrated more obvious degenerative adjustments supplemented by intense leukocytic infiltration (Fig. 4A, B). The underlying connective tissue stroma was invaded by blood vessels alongside severe edema (Fig. 4C, D), deposition of more abundant and thicker, blue-stained collagen bundles beside ciliary body hyperplasia, and inflammatory cells infiltration (Fig. 4E, F).

**Fig. 5:**
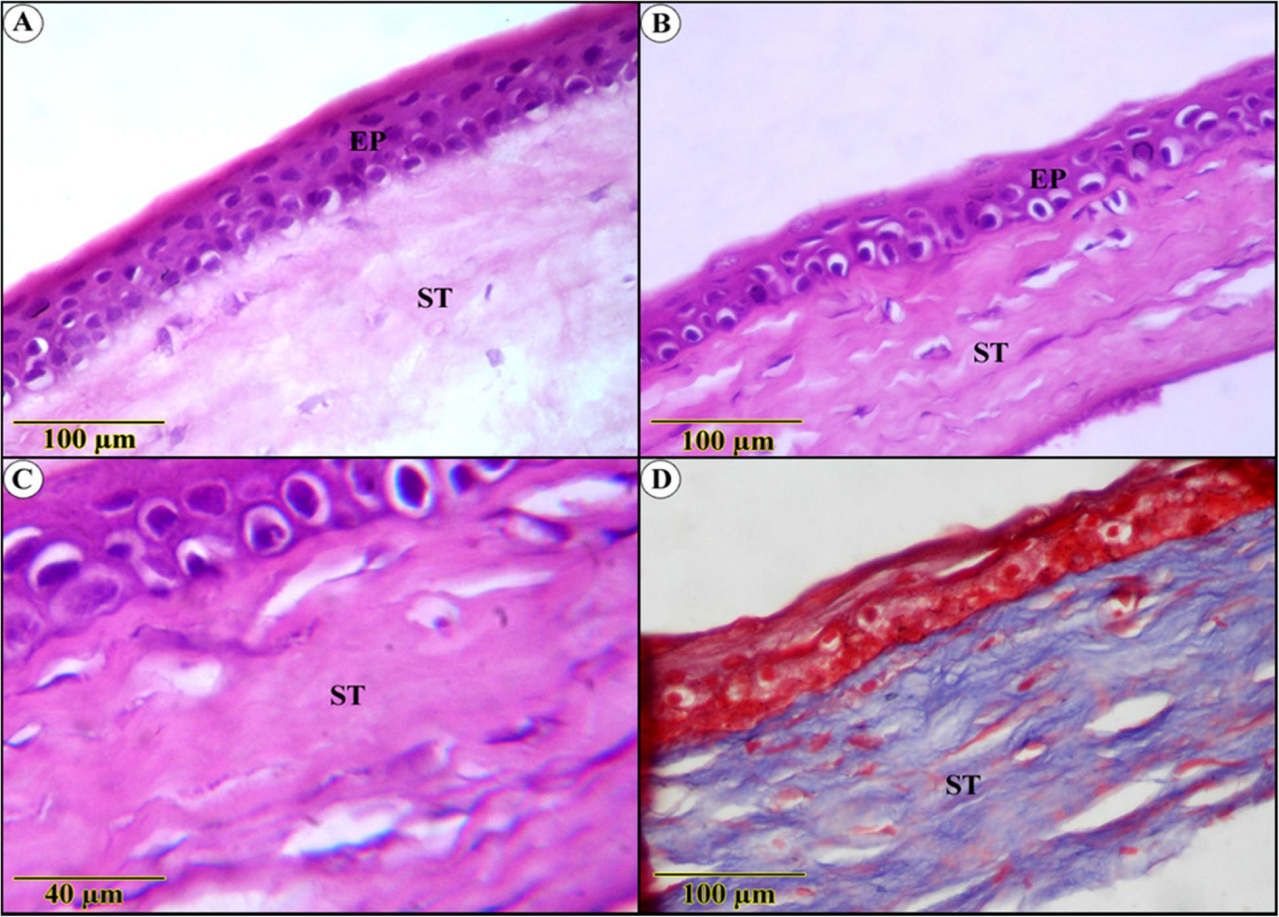
Histological sections of the cornea of pycnogenol effect on diabetic-ulcerated rats (**A**, **B**, **C**, and **D**) show a normal structure of corneal epithelium, supported by Bowman’s membrane (BB). Regularly arranged acidophilic and blue stained stroma and Descement’s membrane (DM)

Apart from Bowman’s membrane restoration, the PYC-effect on diabetic-ulcerated rats was striking, with the regeneration of the epithelium and fibrotic stroma approaching normal architecture (Fig. 5A). In addition, the collagen bundles appear ameliorated with a highly noticed organized arrangement, as opposed to slightly noticed edema (Fig. 5D). The Descemet’s membrane and endothelium were intact in most corneal parts. The epithelium still has vacuolated cytoplasm with darkly stained nuclei in most parts (Fig. 5B, C).

## Discussion

Alkali burns of the cornea are among the most severe acute ocular wounds. Secondary difficulties, i.e.*,* perforation, infection, neovascularization, ulceration, and opacification, can acquire and menace the patients’ vision. Therefore, timely and operative treatment is required to improve and maintain the biological healing handled by several medical means [3, 18]. Compared to the control group, topical phenytoin 1% improved the repair of epithelial defects produced by alkali burns in rabbits [19]. Therefore, the topical functions of PYC in the current study facilitated the CWH in an in vivo (CA-I) model in diabetic rats. The increased CWH was associated with quick re-epithelialization and decreased inflammation. The issue that add amerit to the current study over the other similar studies.

Corneal injury stimulated by an alkali burn typically takes longer to heal than wound finality than mechanical de-epithelization. This variation is mainly because an alkali burn elicits stromal dysregulated inflammation and scar creation by stimulating immune cell infiltration and myofibroblast creation from the keratocytes. This kind of inflammatory retort is not self-restricting and is related to the stromal and epithelial dilapidation [1]. These pathological results were the primary motivation behind the selection of the NaOH alkali burn model to estimate the effects of PYC on CWH. Several treatment modalities have been estimated, containing amniotic membrane grafting [11], sub-conjunctival addition of platelet-rich plasma, topical functions of acetylcysteine [20], honey, umbilical cord serum, propolis extract, and Na-hyaluronan [21]. As previously stated [14], the group treated with PYC reduced wound size the most when compared to the untreated wounds in healthy and diabetic rats.

Hyperglycemia is a symptom of diabetes mellitus. Even though hyperglycemia can be managed with insulin or antihyperglycemic drugs, diabetic retinopathy remains the leading cause of blindness since oxidative stress may play a role in the development of retinopathy. The glutathione peroxidase activity was reduced after treatment with pycnogenol in diabetic rats [22]. PYC mainly consists of phenolic acids and procyanidins, which are used worldwide as an herbal therapy for wound healing. Previous findings have found that it quickens the wound healing processes, mainly due to its anti-inflammatory and wound healing activities. The decline in wound size is subsequent to re-epithelialization [23]. In the current study, regardless of whether the rats were diabetic or not, topical application of PYC accelerated corneal epithelial wound closure. These findings are in agreement with [19, 24]. On contrary, the rate of healing in wounds treated with PYC in rats was faster than in the control group. However, it was unable to induce additional positive activities in diabetic rats [14].

CWH is a multifaceted process requiring various tissues, growth factors, cytokines, and cell lineages. Re-epithelialization is an acute conducive process that promotes successful corneal healing. Suspended corneal re-epithelialization is difficult for diabetes, raises the infection risk, and is related to the heightened inflammation and inadequate stromal modification, causing a transparency loss of corneal tissues [4]. In the current study, the healing rate of DPYC was significantly greater than in the DC group throughout the 6 d of the study period. These findings are inconsistent with [19].

In contrast, the gradual interval in the healing progression of the ocular exterior epithelium was time-dependent regarding the diabetic state. In the current study, the healing process in the diabetic rats treated with the PYC appeared matching to that in non-diabetic rats treated with PYC throughout the 6 d of the study period. This finding could be attributed to the ability of PYC to decrease the oxidized ascorbate and may prolong the vitamin activity in the wound vicinity, sustaining the collagen fabrication. The stated binding of PYC to elastin and collagen has a significant repressing action on the matrix metalloproteinases (MMPs), which may play a significant role in wound healing [16]. The presence of a higher level of proteinases in the wound fluid has been correlated with delayed wound healing [5]. Additionally, PYC has been recently discovered to have bacteriostatic effects versus a broad spectrum of G-positive, G-negative bacteria and *Candida Albicans*. Hence, additional reports of the impacts of PYC in corneal re-epithelialization after alkali burn in furthered state of diabetes are demanded before the final recommendations can be made.

Histologically, the ulcer-induced sections showed distinguished erosion in the epithelial layer with disheveled cellular composition. This intense distortion was after lipophilic Alkali mediators penetrated corneal stroma and torn down proteoglycan ground substance and collagen bundles, resulting in the secretion of proteolytic enzymes and further impairment [25]. This alteration could also be attributed to cytokine oxidative derivatives that could cause direct and indirect changes in the permeability of capillaries and venule blood–tissue barriers, increasing their hydrostatic pressure [26]. The consequences, as mentioned above, combined with the severe interruption of Descement’s membrane, lead to the inflow of aqueous humor into the cornea, with subsequent stromal edema [27]. One of the basic features of edema is the semi-regular separation of structural elements, whereas the keratocytes are separated from adjacent collagen fibers, which also move apart from one another. These findings, consistent with vasculitis, clearly validate the stromal edema and exclude the existence of the histological artifacts. This excessive stromal matrix accumulation and corneal decompensation interfere with light transmission, and, hence, with cornea’s transparency, they are considered the primary causes of opacity appearance in the macroscopic figures [28]. Upon erosion of the corneal epithelium, the basement membrane faces proteases-releasing inflammatory cells that cause degradation of basal membrane structures and interruption of corneal endothelial cells, which have a significant role in normalizing corneal hydration [29].

Consequently, both the basement and Descement’s membrane amend their function and structure due to this massive decomposition. Even though the cornea is one of the few tissues which actively sustain an avascular state, defects in the corneal epithelium precede stromal degradation and, subsequently, neovascularization to aid in healing and untransparent scar formation. This neovascularization is adequate for the severity and extent of the inflammatory intense and its extent. The delay in diabetic corneal epithelial wound-healing could be the main reason behind the severity of degenerative changes that invade the tissue. This delay may be due to the abnormal addition of the basement membrane to stroma and fluctuations in the hemidesmosome realization sites related to this situation [30, 31]. It was reported that throughout prolonged hyperglycemia, glucose cause auto-oxidation of proteins and induces the production of reactive oxygen species (ROS [32];. Therefore, it decreases antioxidant defense and enhances oxidative stress in cells; as a reverse reaction of these alterations, corneal epithelial is subjected to dynamic transformation to initiate a complex healing process. The complexity of this process is due to the incessant proliferation and differentiation of basal epithelial cells, endothelial cells migration, limbal stem cells conversion, extracellular matrix remodeling, and keratocytes diversion to myofibroblasts by the enhancement of growth factor-b (TGF-b) system [28].

Numerous antioxidants have been recently utilized for their protective effect against pro-oxidant that induce oxidative stress either by generating ROS and RNS or by inhibiting antioxidant systems. PYC is a potent anti-oxidizing agent with a reputation for scavenging ROS and reactive nitrogen species (RNS); it can double the intracellular synthesis of anti-oxidative enzymes and behave as a potent devourer to free radicals [16]. Furthermore, they have the ability to inhibit the representation of the proinflammatory cytokine interleukin-1 by adjusting redox receptive transcription factors. Recently, it has been noted that PYC may interact with the blood vessel wall and generate a capillary ‘sealing’ effect, resulting in reducing edema and capillary absorbency [17]. PYC has shown to have promising results in improving conditions, including diabetes [16, 33]. These effects manifested in our result, which demonstrated notable signs of the highly proliferative activity in the stromal lamellae and conspicuous regression of immature collagen fibers proofing the retraction in proinflammatory cytokine.

### Limitations

The current study’s limitations must be acknowledged. First, the small number of rats may cause bias and thus an imprecise conclusion. Secondly, this research only dealt with the histopathological and macroscopical alterations; hence additional investigations should link the macroscopical and microscopical alterations with inflammatory blood markers. Third, a shortage of information about the mechanism by which PYC enhances the deficiency in wound healing related to diabetes may also influence the interpretation of our findings. Therefore, additional cellular and molecular analyses are required to simplify the mechanism. All limitations of our study should be measured in additional studies to have an actual conclusion.

## Conclusion

Clinical and histological outcomes revealed that the topical application of PYC substance showed a substantial improvement in CWH rate in both normal and diabetic rats. At the histological level, facilitated re-epithelization and inflammatory-reduction processes accompanied by the highly noticed organized arrangement of collagen bundles that almost approaches the normal architecture confirmed the ability of the substance to ameliorate the corneal recovery process. At microscopical levels, no signs of adverse reactions were detected, indicating the safety and efficiency of the remedy. Therefore, PYC could be considered an efficient and innocuous drug for promoting CWH in diabetic keratopathy.

## Methods

### Animals

Seven-week-old male Wistar rats (*N* = 28) weighing 150–200 g were utilized in our current study. All the rats were handed a period of 14 d to adapt to the surroundings before beginning the experiment and accommodated under standard laboratory circumstances. All rats received pelleted feed and tap-H2O ad libitum and were accommodated at regulated ambient temperature (22.0 ± 1.0 °C) under a 12-h/12-h dark plan. All experimental procedures at Mansoura University were approved by the Ethics and Animal Experiments Committee.

Diabetes was induced by a single intraperitoneal (i.p.) injection of 55 mg/kg Streptozotocin (STZ, Sigma-Aldrich, Cairo, Egypt) in ice-cold 0.1 M citrate buffer (pH 4.5). Another group of animals only obtained citrate buffer and were measured as normal. Blood glucose levels were observed from the tail vein using an automated blood glucose analyzer (Glucometer Elite XL; Bayer Corporation, Elkhart, IN, USA) instantly prior to obtaining STZ and at 1 wk. post-STZ-insertion. Diabetic rats had blood glucose levels greater than 300 mg/dl. During the experiment, blood glucose levels in STZ-rats remained fourfold higher than in control rats.

### Experimental study

Two weeks post-insertion of STZ, all rats were anesthetized with a blend of ketamine (80 mg/kg) and xylazine (7 mg/kg) i.p. Certified veterinary ophthalmologist performed corneal wounding. In all rats, the mid cornea of the right eye was hurt by locating a filter paper (round 3.0 mm diameter) satiated with 0.01 M NaOH on it for 45 s, then 0.9% physiological saline was used to wash the wound surface. The rats were assigned into four groups (7 rats each) as mentioned: NC, NPYC, DC, and DPYC. Topical soluble phenytoin 1% was prepared by dissolving powder (20 mg phenytoin) (Air Green Co., Lid., Fukui, Japan) with sterile distilled water. The PYC-treated groups received PYC eye drops as an individual drop (0.05 mL) to the mid cornea of the injured eye three times daily for 6 d, and the NC-groups were remedied with vehicle (saline) eye drops in the same means as described [19]. Eye drops were released to the unanesthetized rats. Any rat that found infection was excluded from the current study. These trials were conducted in a double-blind manner to elude any bias.

Corneal wound sizes were measured by staining the ocular exteriors with 1% of fluorescein sodium, then immediately snapped after injury (time 0), 2-, 4-, and 6-d post wounding. The wounds’ areas were measured from photographs using NIH Image J analyzer software (http://rsbweb.nih.gov/ij). The percentage of wound healing was expressed by the following formula: Wound healing (%) = 100 × (wound range on 0-d 0 – wound range on x-d) / wound range on 0-d.

### Histological processing

Six days after wounding, animals were euthanized with sodium pentobarbital i.p. (.100 mg/kg), decollated, and the eyes proposed and enucleated.

For achieving the histopathological evaluation of both treated and injured groups, the globe of both eyes was enucleated with a careful evaluation of vitreous fluid, and elimination of the lens was considered. The specimens were immediately fixed in 10% neutral buffered formalin night long, and then they were processed for the paraffin embedding technique. The paraffinized eye blocks were sectioned using a rotatory microtome into 5 μm thick adhered serial sections (ribbon). This ribbon was mounted onto glycerol-albumin-coated glass slides and stained by Harris’ hematoxylin and eosin stain (H & E) and Trichrome stain.

### Statistical analysis

Data were expressed as mean ± SD. The defect area was analyzed at each time point via analysis of variance and Newman–Keuls tests, where *P* < 0.05 was measured as statistically significant. The statistical analysis was done with software (GraphPad Prism for Windows, version 5.0; GraphPad Software Inc., San Diego, CA, USA).

## Data Availability

The datasets generated during and analyzed during the current study are available at the following link; https://docs.google.com/spreadsheets/d/160IFpt9hE3gYlbrwvmxQmcV62QVo5Ph3/edit?usp=sharing&ouid=117094566402563259268&rtpof=true&sd=true.

## Abbreviations

DM: Diabetes mellitus
CWH: Corneal wound healing
CA-I: Corneal alkali-burn injury
NC: Normal control
NPYC: Normal PYC
DC: Diabetic control
DPYC: Diabetic PYC
